# Owning the Trauma Bay: Teaching Trauma Resuscitation to Emergency Medicine Residents and Nurses through In-situ Simulation

**DOI:** 10.21980/J8WK9X

**Published:** 2020-10-15

**Authors:** Andrew Bellino, Alexandra June Gordon, Al’ai Alvarez, Kimberly Schertzer

**Affiliations:** * Stanford University School of Medicine, Department of Emergency Medicine, Stanford, CA; ^ Stanford University School of Medicine, Department of Critical Care Medicine, Stanford, CA

## Abstract

**Audience:**

The following two cases were designed to address learning objectives specific to interns, junior residents, and senior residents in emergency medicine, as well as trauma-certified emergency nurses.

**Introduction:**

Traumatic and unintentional injuries account for 5.8 million deaths across the globe each year, with a high proportion of those deaths occurring within the initial hour from the time of injury. This “golden hour” begins in the pre-hospital setting yet is predominantly spent in the emergency department (ED).^1^ Being able to effectively manage the multidisciplinary team required to care for trauma patients is crucial to providing timely and appropriate care. In-situ simulation, where the learning case is moved out of the simulation lab and into the typical workplace, has emerged as a powerful training tool for improving care-systems and team dynamics.^2^,^3^ Multiple specialties have shown in-situ simulation to be an effective strategy to teach both educational content as well as critical procedural and communication skills.^4^,^5^ In-situ simulation training has also been applied with similar success to trauma management, allowing for the simultaneous education of different learners with different roles in trauma resuscitations.^6^,^7^ We present two in-situ simulation cases with specific educational objectives and feedback mechanisms that allow for easy implementation of a cost-effective approach to training multidisciplinary emergency medicine providers in trauma management.

**Educational Objectives**: The core objectives of these simulations center on effective teamwork and communication during trauma resuscitation of a critically ill patient. Both cases are designed to include maneuvers that require coordinating team members’ actions during a stressful situation such as rolling a vomiting patient with a head injury and applying a binder to an unstable pelvic fracture. While the cases are largely focused on improving communication, salient learning points on emergent management of intracranial hemorrhage and unstable pelvic fractures are highlighted during the encounter. In addition, this simulation module allowed for the practice of graduated level of responsibilities amongst residents in the trauma bay.

**Educational Methods:**

Two in-situ simulation cases were run with the same group of learners using standardized patient actors as patients and functional medical equipment in actual rooms in the emergency department to recreate a realistic experience. These groups were composed of emergency medicine residents with at least one intern, one junior resident, and one senior resident in each group as well as a bedside nurse, documenting nurse, and simulation instructor. Each case was followed by a group debriefing session using multiple sources of feedback. Standardized patients, bedside nursing, and simulation instructors were all incorporated into the feedback and debriefing process.

**Research Methods:**

Pre- and post-simulation surveys were given to participants to assess their confidence in participating and leading trauma resuscitations.

**Results:**

A total of 29 emergency medicine residents completed both our pre- and post-survey. We found that less than half of those surveyed felt comfortable leading trauma resuscitations. After the simulation scenarios, an overwhelming majority agreed that they felt more prepared to run trauma resuscitations as a result of the simulation experience. In their free response comments participants also remarked upon the ability of in-situ simulation to better foster realistic learning opportunities with regards to communication and resuscitation management.

**Discussion:**

Based on our survey results, we found that a large portion of our participants did not feel comfortable leading trauma resuscitations. The post-survey and the free-text responses collected during the case scenarios show that our in-situ simulation proved to be an effective way to teach various types of learners new trauma roles and optimize high-stress communication during resuscitations. The use of in-situ simulation provides an effective and easily adapted framework even for those outside of academic centers and simulation labs while also offering an opportunity for multidisciplinary growth. Regular incorporation of similar learning opportunities into resident, nursing, and staff education can lead to better communication and teamwork during in-vivo patient encounters.

**Topics:**

Trauma resuscitation, in-situ simulation, code leader education, communication training.

## USER GUIDE

**Table:** 

List of Resources:	
Abstract	108
User Guide	110
Case 1: Instructor Materials	113
Case 1: Operator Materials	123
Case 1: Debriefing and Evaluation Pearls	135
Case 1: Simulation Assessment	127
Case 2: Instructor Materials	132
Case 2: Operator Materials	139
Case 2: Debriefing and Evaluation Pearls	142
Case 2: Simulation Assessment	144

**Learner Audience:**

Emergency Medicine junior residents, senior residents

**Time Required for Implementation:**

Instructor Preparation: 45 minutes to arrange rooms and organize equipment on the day of simulation. Obtaining proper equipment, training volunteers, and coordinating timing and location can take many days depending on each group’s familiarity with in-situ simulation. It is best to start weeks in advance as a lot of the fidelity of in-situ simulation hinges on proper preparation.

Time for case: 15 minutes (Case 1); 15 minutes (Case 2) Time for debriefing: 20 minutes (Case 1); 25 minutes (Case 2)

**Recommended Number of Learners per Instructor:**

Five learners total, ideally one intern as survey physician, one junior resident as procedures/airway physician, one senior resident as code leader, one bedside nurse, and one documenting nurse.

**Topics:**

Trauma resuscitation, in-situ simulation, code leader education, communication training.

**Objectives:**

By the end of this simulation session, the learner will be able to:

1. identify and learn to incorporate aspects of good resuscitation leadership including role designation, closed-loop communication, and crowd control,
2. recognize changes in patients’ vital signs and mental status and appropriately escalate interventions,
3. understand emergency management of traumatic intracranial hemorrhages including anticoagulation reversal, airway management, and decreasing intracranial pressure, and
4. stabilize pelvic fractures using a binder and understand the proper use of a massive transfusion protocol and embolization in unstable pelvic trauma victims.

### Linked objectives and methods

Effective trauma resuscitations require a well-choreographed team approach with the code leader coordinating tasks, listening to data transmitted by the person performing the trauma survey, ensuring adequate hemodynamic monitoring and establishing appropriate intravenous access, all while ensuring the entire process is streamlined to allow the documenting nurse to hear and properly record events (objective 1). This complex series of coordination can easily distract from subtle clues that the patient is decompensating, which the learner should strive to overcome by remaining vigilant to vital signs and rapid alterations in mental status (objective 2). These cases also include maneuvers that require coordinating multiple members of the team to work together. This includes rolling the patient (objective 1), airway management (objectives 1 and 3), and the application of a pelvic binder (objectives 1 and 4). Case 1 is an encounter of a patient on anticoagulation with a traumatic intracranial hemorrhage requiring learners to discuss and implement reversal of anticoagulation as well as emergent measures to decrease intracranial pressure (objective 3). Case 2 is an unstable open-book pelvic fracture and requires learners to identify the need for a massive transfusion protocol and involvement of the interventional radiology team for emergent embolization (objective 4).

### Recommended pre-reading for instructor

Instructors should be advanced trauma life support (ATLS) certified and be familiar with basic emergency management of acute head trauma and pelvic trauma (including placement of a pelvic binder). Textbook chapters and society guidelines specific to these cases are found in References 8–12 for further reading. Instructors should also be familiar with effective code leader skills such as role clarity, closed-loop communication, and crowd control. Instructors wanting to review any of these topics can find more information in Reference 13.

### Learner responsible content

Learners should be familiar with ATLS and basic trauma management, but no specific pre-reading is required. If learners have not had much education on effective resuscitation communication strategies, we recommend reviewing Reference 13.

### Associated content (optional)

We have included the form we used for nurse documenters to evaluate the team.

### Results and tips for successful implementation

We performed both a pre- and post-survey of the emergency medicine residents who participated in the simulations, with a total 29 respondents. The pre-survey asked participants to assess their level of comfort leading trauma resuscitations, with 19% “very comfortable,” 26% “slightly comfortable,” 14% “neither comfortable nor uncomfortable,” 5% “uncomfortable,” and 36% “very uncomfortable.” On the post-survey 45% stated that they “Strongly Agreed” that the simulations had helped prepare them to be better able to run trauma resuscitations, while another 45% “Slightly Agreed” with the statement. There were 7% of participants who were “Neutral” to the statement and 3% who “Disagreed.” When we analyzed the free text comments, we noted several themes. Two of the 23 comments indicated that the participants preferred the in-situ format to the traditional simulation center. Another two of 23 comments referenced systems issues they learned or reinforced during the simulations. Participants felt more confident or prepared after having participated in these simulations (4 of 23 comments), and the most commonly mentioned feedback involved having a better appreciation for the team captain role (5 of 23 comments). Even those who did not act as team captain felt they had “some insight into role delegation and relative responsibilities.”

We were encouraged by these results and believe the scenarios and learning objectives are best achieved when utilized with a group of varying levels of training. We used a PGY-1 resident to perform the trauma survey examination, a PGY-2 resident to perform procedures/point of care ultrasound, a PGY-3 resident to serve as the code leader, a trauma-certified bedside nurse, and a registered nurse to handle documentation similar to real-time ED workflow. Additionally, if present at your institution, an emergency department pharmacist can be recruited to help dispense medications during the scenarios and discuss the pros and cons of different medical management for increased intracranial pressure in case 1. The case scenarios allow for junior residents (PGY-1 and PGY-2) to perform trauma management (eg, objectives 3 and 4) directed by the senior resident (PGY-3). The case scenarios also allow for optimizing communication delivery between senior physicians and nurses during resuscitations with a specific objective of improving teamwork skills (eg, objectives 1 and 2). Ultimately, the debriefings allow for all involved learners to be exposed to all of the learning objectives for different levels without feeling overwhelmed in each of their respective roles. In our module, we partnered with nursing leadership to provide nursing-specific trauma continuing education. The modules met nursing-specific educational goals such as managing the rapid fluid infuser, which allowed for increased nursing participation. We utilized a specific feedback form for nursing documenters to increase sources of evaluation and augment debriefing (see associated content).

An added benefit of using in-situ simulation rather than a simulation lab was that we were able to perform both cases back to back in five different rooms simultaneously in an ED zone prior to daily opening during the resident weekly educational conference. All residents went through the learning objectives at the same time. We were then able to hold a large group debriefing session after the individual case debriefs, which allowed each group to share their main learning points. Based on our post-session surveys, both nurses and residents enjoyed the active and interdisciplinary learning as well as the in-situ format, which had not been used frequently in their educational conference up to that point. Additionally, nursing staff did not traditionally attend the resident education conference and this provided an opportunity to bring their perspective to resident education.

While this format did bring its own advantages, we realize that for many it is not feasible to always count on a specific area of the emergency department to be empty or to have all residents free at the same time. One alternative option to the location is to consider using a simulation room or simulation center, but to keep the equipment used during the simulation as close to that encountered in your emergency department as possible such as real patient actors, backboards, ultrasound machine, and procedural equipment. Another possibility is to use a conference room or classroom, again with as much real-life equipment as possible, because the educational benefit will still be achieved from the interpersonal interactions and troubleshooting required by the cases. If all residents are not available at the same time, or there is not a space large enough to accommodate them all at once, the scenarios can be performed by one group at a time and then rotated, either throughout the same day, or while performing other group learning activities, or across multiple weeks. Additionally, filming each group’s simulation can be a way to highlight key takeaways and educate residents or nurses who may not be able to make it in person.

## Supplementary Information











## Figures and Tables

**Table:** 

#1	Normal chest radiograph (CXR)
#2	Normal pelvic radiograph (PXR)
#3	Computed Tomography (CT) axial slice showing epidural hematoma
#4	Chest radiograph (CXR) showing endotracheal tube in the appropriate position
#5	Case 1 lab values

**Table:** 

**Vitals:**	Heart Rate (HR) 76	Blood Pressure (BP) 120/80	Respiratory Rate (RR) 16
	Temperature (T) 37°C	Oxygen Saturation (O_2_Sat) 98% on room air	

**Figure:**
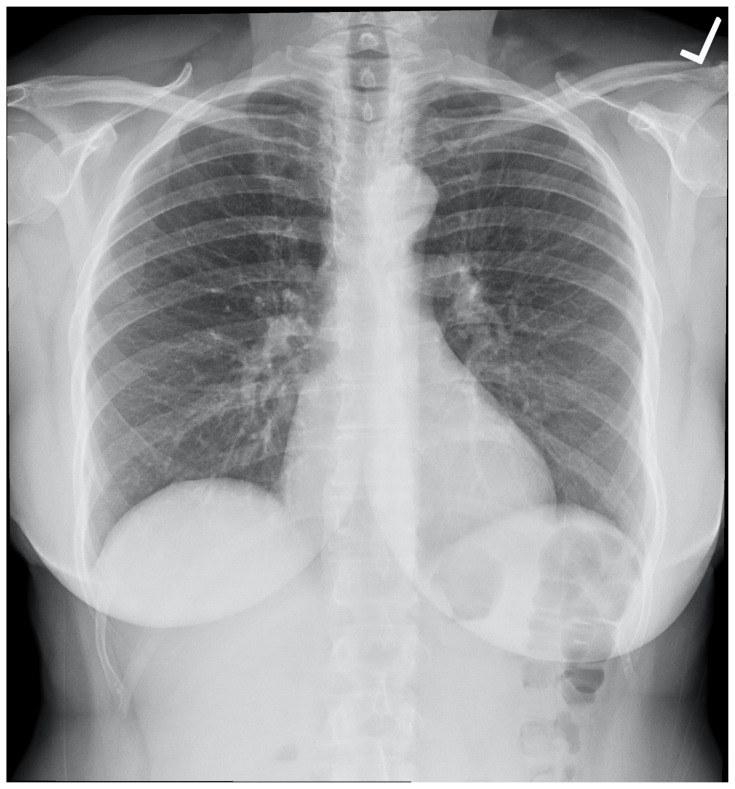


**Figure:**
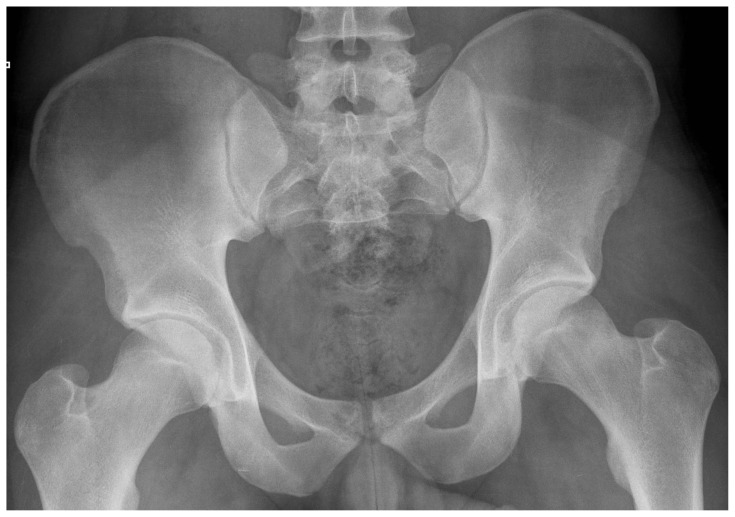


**Figure:**
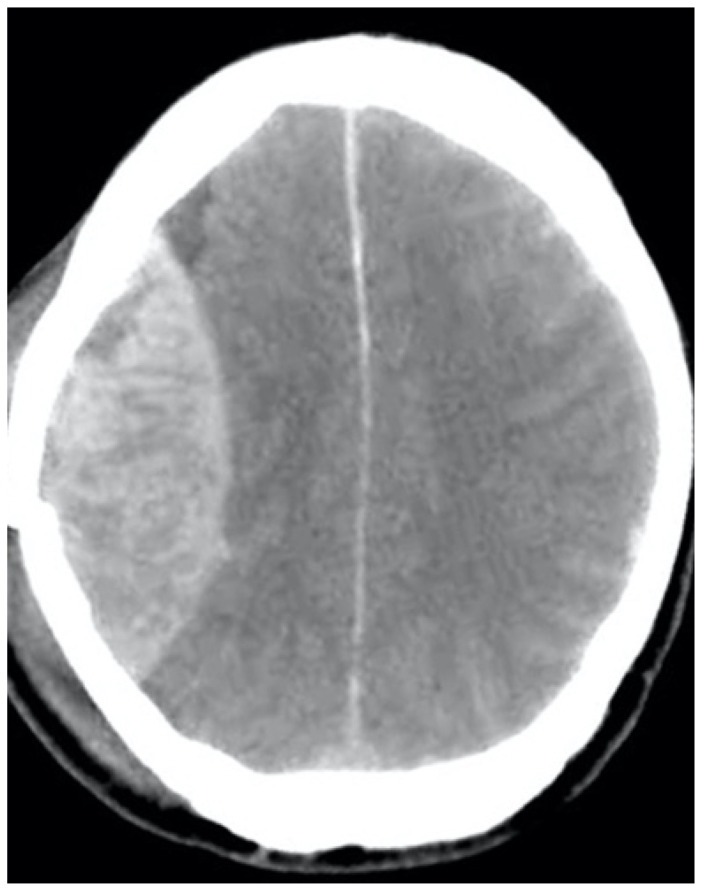


**Figure:**
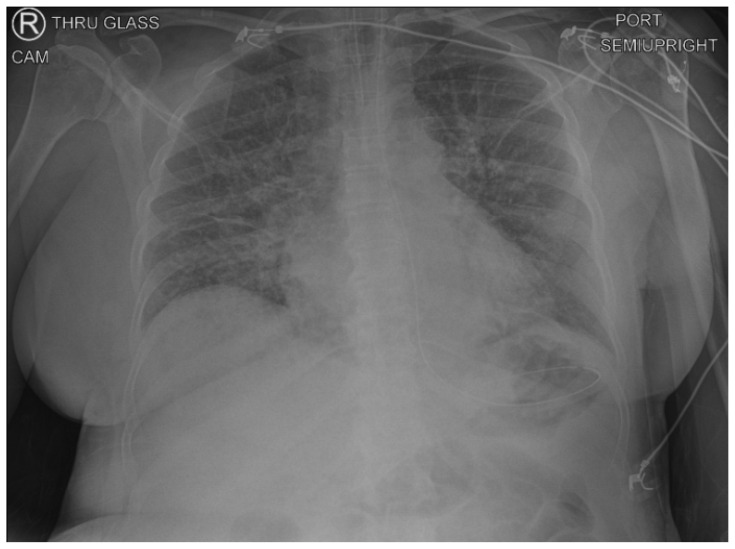


**Table:** 

*Complete blood count (CBC)*	
White blood count (WBC)	16.0 × 1000/mm^3^
Hemoglobin (Hgb)	10.0 g/dL
Hematocrit (HCT)	30.0%
Platelet (Plt)	130 × 1000/mm^3^

**Table:** 

*Basic metabolic panel (BMP)*	
Sodium	138 mEq/L
Potassium	3.6 mEq/L
Chloride	110 mEq/L
Bicarbonate (HCO_3_)	14 mEq/L
Blood Urea Nitrogen (BUN)	30 mg/dL
Creatinine (Cr)	1.2 mg/dL
Glucose	125 mg/dL

**Table:** 

*Liver Function Test (LFT)*	
Aspartate Aminotransferase (AST)	45 U/L
Alanine Aminotransferase (ALT)	35 U/L
Total bilirubin	0.6 mg/dL
Albumin	3.5 g/dL
Alkaline Phosphate	110 U/L

**Table:** 

Prothrombin Time/International Normalized Ratio (INR)	5.8

**Table:** SIMULATION EVENTS TABLE:

Minute (state)	Participant action/ trigger	Patient status (simulator response) & operator prompts	Monitor display (vital signs)
0:00 (Baseline)	The team arrives in a room with the patient on the backboard already, and EMS begins giving a report. Allow time for the team to ask questions to EMS, after which the EMS team leaves and does not return. If team asks specifically about medications, EMS hands one member the bag of pill bottles; if team does not ask, they leave the bag on the patient’s lap. The primary survey begins and the bedside nurse should start simulating IV placement.	If the patient is questioned, he or she responds but in a way that does not answer the question. The patient appears calm with no complaints	Wait to display vitals until someone simulates placing the patient on the monitor. T 37C HR 76 BP 120/80 RR 16 O2 98% If learners obtain a manual BP prior to placing on the monitor, verbally give the BP reading.
0:45	The primary survey should be complete. The patient should be on the monitor. Procedure physician should be performing FAST on the standardized patient (normal)	If the patient has not yet been put on the monitor, have the documenting nurse ask if they should get vital signs. If not performing FAST (Focused Assessment with Sonography for Trauma) can omit in this case; however, using it adds to team coordination.	Same as above.
2:00	Roll the patient to inspect the back. If team has not identified medications by this point, bedside RN grabs bag prior to roll and asks a resident to look through it.	The patient complains, and groans when rolled. Note practice variations: backboard may or may not be removed during this time.	Same as above.
2:20	Roll the patient back to the supine position.	The patient begins to groan and gag. If not rolled again onto the side, the patient begins to vomit on self.	HR 110 BP 150/90 RR 18 O2 96%
3:00	Patient suctioned, stops vomiting, and is rolled back to the supine position.	The patient now has a GCS score of E1V2M3 (GCS6). The team should decide to intubate. If they do not intubate, the patient continues to gag.	Same as above. If no intubation O2 drops to 60%, HR increases to 130. If team does not notice deteriorating vital signs, documenting RN or instructor can voice them out loud.
7:00	Patient intubated (medications called for and given by RN, ETT taped to the side of the face).	Paralyzed. If short-acting paralytic is used (succinylcholine) and no sedation ordered, the patient begins to have jerking movements.	If no sedation ordered patient HR increases to 160, BP 180/100
11:00	Call for labs including INR. Order the reversal of anticoagulation. Send the patient to the CT scanner.	INR results at 5.8, can consider reversal with four-factor prothrombin complex concentrate at this time or wait until after CT scan. The patient should already be sedated, intubated. The team captain should indicate that RT, nursing staff, and a physician should accompany the patient to CT (patient does not have to physically leave room for CT, instructor can announce “patient goes to CT and returns without incident”).	Same as previous.
(End of the case)	Instructor hands team leader CT image showing epidural hematoma.	The team should discuss management of increased ICP, including anticoagulation reversal and giving mannitol or hypertonic saline. Neurosurgery should be consulted, and RN can prompt for consult if not done by team. If not managing increased intracranial pressure or have not reversed anticoagulation, instructor can prompt for this (can assume role of neurosurgery consultant, if desired). Case ends once ICP is effectively managed.	If no medications are given for increased ICP: HR 35 BP 200/110

**Table:** 

**Critical Actions:** Roll patient and suction to avoid aspiration when vomiting Recognize declining GCS and decide to intubate Obtain CT of head Obtain INR and reverse once given result Give either hypertonic saline or mannitol to reduce intracranial pressure	0:00

**Table:** 

	Milestone	Did not achieve level 1	Level 1	Level 2	Level 3
1	**Emergency Stabilization (PC1)**	□ Did not achieve Level 1	□ Recognizes abnormal vital signs	□ Recognizes an unstable patient, requiring intervention Performs primary assessment Discerns data to formulate a diagnostic impression/plan	□ Manages and prioritizes critical actions in a critically ill patient Reassesses after implementing a stabilizing intervention
2	**Performance of focused history and physical (PC2)**	□ Did not achieve Level 1	□ Performs a reliable, comprehensive history and physical exam	□ Performs and communicates a focused history and physical exam based on chief complaint and urgent issues	□ Prioritizes essential components of history and physical exam given dynamic circumstances
3	**Diagnostic studies (PC3)**	□ Did not achieve Level 1	□ Determines the necessity of diagnostic studies	□ Orders appropriate diagnostic studies. Performs appropriate bedside diagnostic studies/procedures	□ Prioritizes essential testing Interprets results of diagnostic studies Reviews risks, benefits, contraindications, and alternatives to a diagnostic study or procedure
4	**Diagnosis (PC4)**	□ Did not achieve Level 1	□ Considers a list of potential diagnoses	□ Considers an appropriate list of potential diagnosis May or may not make correct diagnosis	□ Makes the appropriate diagnosis Considers other potential diagnoses, avoiding premature closure
5	**Pharmacotherapy (PC5)**	□ Did not achieve Level 1	□ Asks patient for drug allergies	□ Selects an medication for therapeutic intervention, consider potential adverse effects	□ Selects the most appropriate medication and understands mechanism of action, effect, and potential side effects Considers and recognizes drug-drug interactions
6	**Observation and reassessment (PC6)**	□ Did not achieve Level 1	□ Reevaluates patient at least one time during case	□ Reevaluates patient after most therapeutic interventions	□ Consistently evaluates the effectiveness of therapies at appropriate intervals
7	**Disposition (PC7)**	□ Did not achieve Level 1	□ Appropriately selects whether to admit or discharge the patient	□ Appropriately selects whether to admit or discharge Involves the expertise of some of the appropriate specialists	□ Educates the patient appropriately about their disposition Assigns patient to an appropriate level of care (ICU/Tele/Floor) Involves expertise of all appropriate specialists
9	**General Approach to Procedures (PC9)**	□ Did not achieve Level 1	□ Identifies pertinent anatomy and physiology for a procedure Uses appropriate Universal Precautions	□ Obtains informed consent Knows indications, contraindications, anatomic landmarks, equipment, anesthetic and procedural technique, and potential complications for common ED procedures	□ Determines a back-up strategy if initial attempts are unsuccessful Correctly interprets results of diagnostic procedure
20	**Professional Values (PROF1)**	□ Did not achieve Level 1	□ Demonstrates caring, honest behavior	□ Exhibits compassion, respect, sensitivity and responsiveness	□ Develops alternative care plans when patients’ personal beliefs and decisions preclude standard care
22	**Patient centered communication (ICS1)**	□ Did not achieve level 1	□ Establishes rapport and demonstrates empathy to patient (and family) Listens effectively	□ Elicits patient’s reason for seeking health care	□ Manages patient expectations in a manner that minimizes potential for stress, conflict, and misunderstanding. Effectively communicates with vulnerable populations, (at risk patients and families)
23	**Team management (ICS2)**	□ Did not achieve level 1	□ Recognizes other members of the patient care team during case (nurse, techs)	□ Communicates pertinent information to other healthcare colleagues	□ Communicates a clear, succinct, and appropriate handoff with specialists and other colleagues Communicates effectively with ancillary staff

**Table:** 

#1	Normal chest radiograph (CXR)
#2	Chest radiograph (CXR) showing endotracheal tube in the appropriate position
#3	Open book pelvic radiograph (PXR)

**Table:** 

**Vitals:**	Heart Rate (HR) 80	Blood Pressure (BP) 130/80	Respiratory Rate (RR) 18
	Temperature (T) 37°C	Oxygen Saturation (O_2_Sat) 100% on room air	

**Figure:**
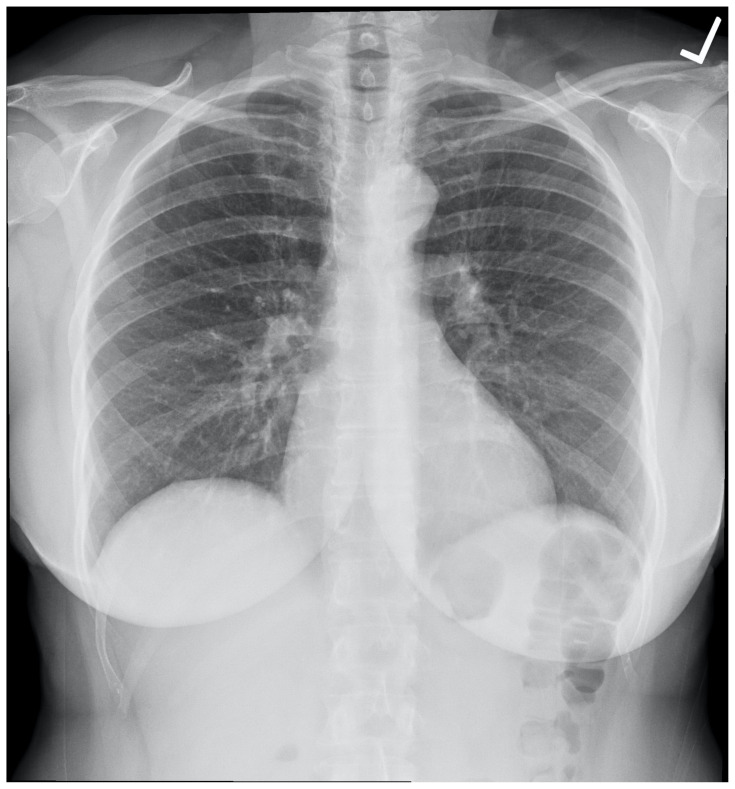


**Figure:**
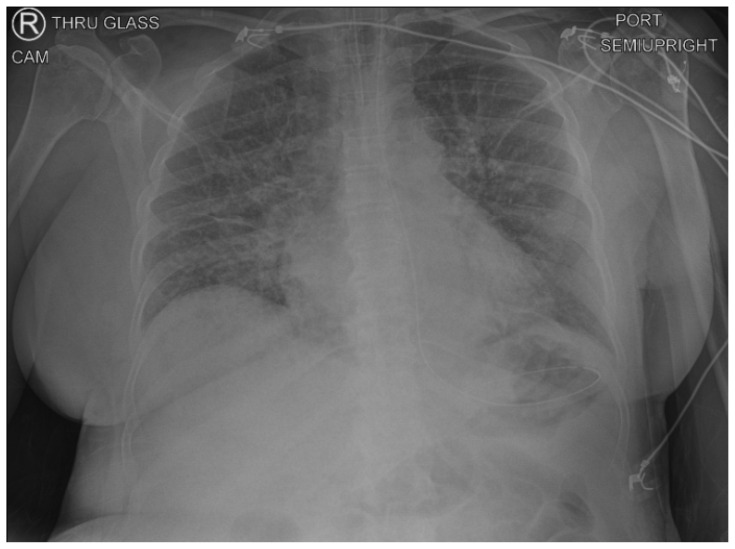


**Figure:**
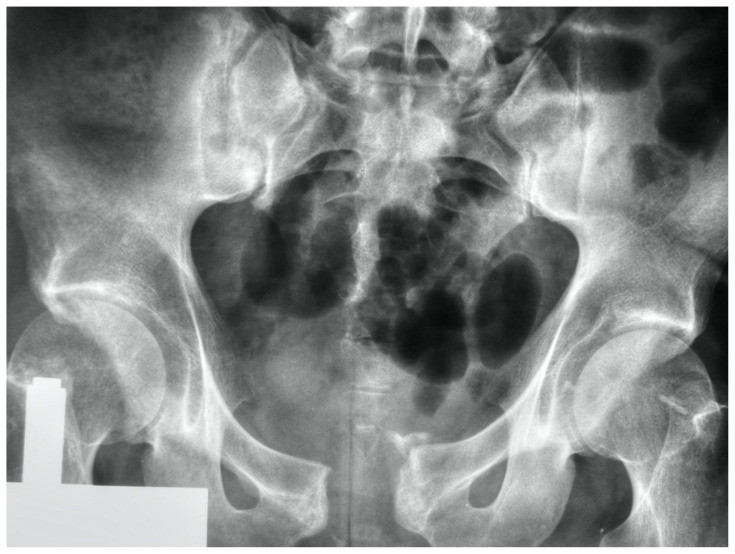


**Table:** SIMULATION EVENTS TABLE:

Minute (State)	Participant Action/ Trigger	Patient Status (Simulator Response) & Operator Prompts	Monitor Display (Vital Signs)
0:00 (Baseline)	Team arrives in room with patient on backboard and EMS begins report. Leave time for team to ask questions to EMS; then they leave and do not return. Primary survey begins and bedside nurse should start simulating IV placement.	If patient is questioned, they respond appropriately. Complaining of pain all along left side.	Wait to display vitals until someone simulates placing the patient on the monitor. T 37C HR 80 BP 130/80 RR 18 O2 100% If learners obtain a manual BP prior to placing on the monitor, verbally give the BP reading.
0:45	Primary survey should be complete. Patient should be on monitor. Procedure physician should be performing FAST on standardized patient (normal).	If patient has not yet been put on monitor, documenting nurse should ask if they should get vitals. If not performing FAST (Focused Assessment with Sonography for Trauma) have documenting nurse ask if they should be performing one. If they assess for pelvic instability, instructor announces there is movement to lateral compression and scenario skips to 02:10.	Same as above.
2:00	Secondary survey proceeds with patient supine. If team tries to roll patient prior to completing all other aspects of secondary survey, patient asks them to wait.	Patient does not want to move lower extremities during secondary survey due to pain. Has normal exam to palpation and inspection of lower extremities.	Same as above.
2:10	Patient rolled to side (or instability assessed, see above).	Patient screams in pain. Vitals become unstable. If team does not notice vital signs changing, documenting RN or instructor can announce new vitals.	HR 140 BP 75/45 RR 24 O2 97%
3:00	Patient rolled back to supine, should apply pelvic binder.	Patient stops screaming in pain. If team does not discuss applying binder, bedside RN or instructor should prompt by asking what can be done about blood pressure.	Once binder applied: HR 110 BP 85/55 RR 20 O2 97%
3:30	Should order emergency blood (O+/− depending on patient sex).	Groaning. If blood has not been ordered prior to or after binding, instructor or RN should prompt.	After blood arrives: HR 100 BP 95/60 RR 20 O2 97%
8:00	Should be finishing secondary survey if they have not already.	Patient complaining of pain in left side again. If team does not notice blood pressure dropping again, instructor or RN prompts.	HR 130 BP 74/40 RR 22 O2 94%
8:30	Call for massive transfusion protocol.	If massive transfusion not considered, becomes more somnolent and quiet. Team can consider intubation at this point, but if they proceed instructor should prompt them to consider blood pressure before intubation. If they still proceed with intubation while patient is hypotensive the patient codes.	Once massive transfusion started: HR 98 BP 100/75 RR 18 O2 95%
10:00	Massive transfusion started; surveys complete.	Patient groaning, answering in one-word sentences. Team should discuss definitive management (IR embolization). If they do not RN can prompt by asking if patient is going to the OR.	Same as above.
10:30	Decision made to call IR for emergent angiography and embolization. Team should decide to intubate in anticipation of transfer to IR suite and persistent hypotension.	Patient remains with eyes closed and short answers. If team does not discuss intubation, instructor can prompt assuming role of IR attending.	Same as above.
14:00	Patient intubated (ETT taped to side of face), all medications called for and delivered by RN.	Intubated, paralyzed. RN should prompt for sedation orders if not asked for by team leader.	Same as above.
End of Case	Sedation ordered; patient readied for transfer to IR suite.	If no sedation ordered and short acting paralytic given, can begin to jerk until sedation called for. Case ends when patient is properly sedated and instructor announces that IR suite is ready for patient.	Same as above.

**Table:** 

**Critical Actions:** Identify unstable pelvis Apply pelvic binder Recognize need for massive transfusion protocol Intubate patient for declining mental status Arrange for embolization of unstable pelvic fracture	0:00

**Table:** 

	Milestone	Did not achieve level 1	Level 1	Level 2	Level 3
1	**Emergency Stabilization (PC1)**	□ Did not achieve Level 1	□ Recognizes abnormal vital signs	□ Recognizes an unstable patient, requiring intervention Performs primary assessment Discerns data to formulate a diagnostic impression/plan	□ Manages and prioritizes critical actions in a critically ill patient Reassesses after implementing a stabilizing intervention
2	**Performance of focused history and physical (PC2)**	□ Did not achieve Level 1	□ Performs a reliable, comprehensive history and physical exam	□ Performs and communicates a focused history and physical exam based on chief complaint and urgent issues	□ Prioritizes essential components of history and physical exam given dynamic circumstances
3	**Diagnostic studies (PC3)**	□ Did not achieve Level 1	□ Determines the necessity of diagnostic studies	□ Orders appropriate diagnostic studies. Performs appropriate bedside diagnostic studies/procedures	□ Prioritizes essential testing Interprets results of diagnostic studies Reviews risks, benefits, contraindications, and alternatives to a diagnostic study or procedure
4	**Diagnosis (PC4)**	□ Did not achieve Level 1	□ Considers a list of potential diagnoses	□ Considers an appropriate list of potential diagnosis May or may not make correct diagnosis	□ Makes the appropriate diagnosis Considers other potential diagnoses, avoiding premature closure
5	**Pharmacotherapy (PC5)**	□ Did not achieve Level 1	□ Asks patient for drug allergies	□ Selects an medication for therapeutic intervention, consider potential adverse effects	□ Selects the most appropriate medication and understands mechanism of action, effect, and potential side effects Considers and recognizes drug-drug interactions
6	**Observation and reassessment (PC6)**	□ Did not achieve Level 1	□ Reevaluates patient at least one time during case	□ Reevaluates patient after most therapeutic interventions	□ Consistently evaluates the effectiveness of therapies at appropriate intervals
7	**Disposition (PC7)**	□ Did not achieve Level 1	□ Appropriately selects whether to admit or discharge the patient	□ Appropriately selects whether to admit or discharge Involves the expertise of some of the appropriate specialists	□ Educates the patient appropriately about their disposition Assigns patient to an appropriate level of care (ICU/Tele/Floor) Involves expertise of all appropriate specialists
9	**General Approach to Procedures (PC9)**	□ Did not achieve Level 1	□ Identifies pertinent anatomy and physiology for a procedure Uses appropriate Universal Precautions	□ Obtains informed consent Knows indications, contraindications, anatomic landmarks, equipment, anesthetic and procedural technique, and potential complications for common ED procedures	□ Determines a back-up strategy if initial attempts are unsuccessful Correctly interprets results of diagnostic procedure
20	**Professional Values (PROF1)**	□ Did not achieve Level 1	□ Demonstrates caring, honest behavior	□ Exhibits compassion, respect, sensitivity and responsiveness	□ Develops alternative care plans when patients’ personal beliefs and decisions preclude standard care
22	**Patient centered communication (ICS1)**	□ Did not achieve level 1	□ Establishes rapport and demonstrates empathy to patient (and family) Listens effectively	□ Elicits patient’s reason for seeking health care	□ Manages patient expectations in a manner that minimizes potential for stress, conflict, and misunderstanding. Effectively communicates with vulnerable populations, (at risk patients and families)
23	**Team management (ICS2)**	□ Did not achieve level 1	□ Recognizes other members of the patient care team during case (nurse, techs)	□ Communicates pertinent information to other healthcare colleagues	□ Communicates a clear, succinct, and appropriate handoff with specialists and other colleagues Communicates effectively with ancillary staff

## INSTRUCTOR MATERIALS

**Case 1 Title:** Fall on Anticoagulation

**Case Description & Diagnosis (short synopsis):** This is a case of an elderly patient on warfarin who falls down several steps and arrives mildly confused. The patient quickly develops increased confusion and begins to vomit requiring airway suctioning. After suctioning, the patient becomes more obtunded requiring intubation. A computed tomography (CT) of the head reveals an epidural hematoma with mass effect. The learners are expected to reverse coagulopathy secondary to warfarin and manage the increased intracranial pressure.

### Equipment or Props Needed

Simulated patient

Moulage (gauze wrapped around the head)

C-collar

Backboard

Point-of-care ultrasound machine

Simulated monitor

Portable computer for documenter (or equivalent for the specific institution)

Endotracheal tube with stylet

Laryngoscope Yankauer^TM^ suction

### Confederates needed

One emergency medical services (EMS) technician, although this role may be performed by the instructor if desired.

### Stimulus Inventory

**Background and brief information:** The patient arrives on a backboard and with c-collar immobilization performed by EMS to a Level 1 trauma center with one peripheral IV established (taped to arm).

**Initial presentation:** The patient is alert and speaking in full sentences although is mildly confused about the events surrounding the fall. The patient has normal vitals.

**How the scene unfolds:** As the primary and secondary survey progress, the patient becomes more confused and somnolent. After being rolled to assess the patient’s back as part of the primary survey of exposure, the patient feels nausea. Upon rolling back, the patient begins to vomit and gag requiring the patient to be rolled again for oral airway suctioning. At this point, the patient becomes minimally responsive. The learners must recognize the need to secure the patient’s airway. If they do not proceed with intubation, the patient becomes unresponsive and hypoxic until the procedure is accomplished successfully. Learners should articulate the need for anticoagulation reversal and the management of increased intracranial pressure (ICP). The latter may be achieved with either hypertonic saline or with mannitol before or after CT imaging is performed.

### Critical actions

1. Roll patient and suction to avoid aspiration when vomiting
2. Recognize declining Glasgow Coma Scale (GCS) and decide to intubate
3. Obtain CT of head
4. Obtain international normalized ratio (INR) and reverse once given result
5. Give either hypertonic saline or mannitol to reduce intracranial pressure

**Case 1 Title:** Fall on Anticoagulation

**Chief Complaint:** fall down 6 stairs

**EMS Report:** 75-year-old male/female who tripped and fell down 6 stairs. The patient does not recall the event, and a neighbor found the patient on the ground and called 911. (if asked): The point of care glucose is 108. They do not list the medication history, but rather hand over a bag of pill bottles in which there is warfarin, amlodipine, and atorvastatin. The patient has no known allergies.

**General Appearance:** alert

### Primary Survey

- **Airway:** intact
- **Breathing:** equal breath sounds
- **Circulation:** palpable central and peripheral pulses, no active bleeding but dried blood on scalp

### History

- **History of present illness:** none known other than EMS report
- **Past medical history:** hypertension, atrial fibrillation, hyperlipidemia
- **Past surgical history:** none
- **Medications:** warfarin, amlodipine, atorvastatin
- **Allergies:** none
- **Social history:** lives alone, otherwise unknown
- **Family history:** unknown

### Secondary Survey/Physical Examination

- **General appearance:** laying on backboard with no complaints
- **HEENT:**
  ○ **Head:** right temporal hematoma with overlying gauze wrap
  ○ **Eyes:** pupils equal round and reactive to light
  ○ **Ears:** dried blood in the right ear, otherwise normal no hemotympanum
  ○ **Nose:** within normal limits
  ○ **Throat:** within normal limits
- **Neck:** in c-collar, nontender
- **Heart:** normal rate, irregularly irregular rhythm
- **Lungs:** equal breath sounds
- **Abdominal/GI:** nontender, nondistended
- **Genitourinary:** no blood or trauma
- **Rectal:** normal rectal tone (may defer rectal exam)
- **Extremities:** normal strength and sensation, no tenderness to palpation or deformities
- **Back:** no thoracic or lumbar tenderness or step offs
- **Neuro:** initially normal other than amnestic to event
- **Skin:** temporal scalp hematoma, small skin tear on the left forearm
- **Psych:** within normal limits

*Normal Chest Radiograph (CXR)*

Chest x-ray plain film normal. In: Wikimedia Commons. https://commons.wikimedia.org/wiki/File:Chest_x-ray_plain_film_normal.jpg. Published November 20, 2017. Accessed June 22, 2020. CC-BY-SA-4.0.

*Normal Pelvic Radiograph (CXR)*

Häggström, M. X-ray of the pelvis of an 18-year-old male-case 3-anteroposterior. In Wikimedia Commons. https://commons.wikimedia.org/wiki/File:X-ray_of_the_pelvis_of_an_18_year_old_male_-_case_3_-_anteroposterior.jpg. Published March 14, 2018. Accessed June 22, 2020.

*Computed Tomography (CT) axial slice showing epidural hematoma*

Hellerhoff. Epidurales Heamatom. In: Wikimedia Commons. https://commons.wikimedia.org/wiki/File:Epidurales_Haematom.jpg. Published November 1, 2009. Accessed June 22, 2020. CC-BY-SA-3.0.

*Chest radiograph (CXR) showing endotracheal tube in the appropriate position*

Author’s own image

*Case 1 lab values*

## OPERATOR MATERIALS

### Diagnosis

traumatic epidural hematoma with mass effect

### Disposition

to the operating room with neurosurgery

## DEBRIEFING AND EVALUATION PEARLS

### Case 1: Fall on Anticoagulation

**Team Communication** (May use the nurse documenter feedback form (associated content) to help drive these communication and teamwork debriefing points.):

- Ask the group how they felt regarding the communication during the resuscitation? What techniques were effective, and what were not? Pay particular attention to examples of closed loop communication, how the room was directed to proceed through specific tasks, or if anyone was asked to step back or pause if things were getting too chaotic.
- Did everyone understand what was being done during the case? How could the team leader have ensured that everyone was aware of what steps were being taken? This is most often done by the leader taking a pause and summarizing what has happened and what will happen next, especially before critical steps occur.
- Was it clear who was leading the resuscitation? How were the roles identified? Role identification is best done prior to the patient arriving, with the team leader announcing their name and role aloud and asking everyone in the room to do the same. Another helpful tactic is for the team leader to stand at the foot of the bed and not perform any other task during the resuscitation.
- Were the appropriate tasks performed in a coordinated, logical fashion? Why or why not? This can often be difficult during a trauma survey as certain interventions need to be done prior to completion of the survey. As the primary survey moves along from airway, breathing, circulation, disability, and exposure (A-B-C-D-E) any life-saving intervention should be performed once the need is identified and prior to moving on to the next element of the survey. Definitive management of issues identified in the primary survey should take priority before beginning the secondary survey.

#### Medical Management

- epidural hematomas can present with a “lucid interval” during which the patient appears well. However, they can quickly decompensate as the hematoma expands
- elderly head trauma patients need to be monitored closely, especially those on anticoagulation
- vomiting trauma patients should be log rolled onto their side to avoid the risk of aspiration
- care should be taken when intubating head injury patients to maintain in-line cervical spine stabilization and avoid hypoxia and hypotension
- patients on warfarin should have their INR checked when they present as trauma patients
- a life-threatening intracranial bleed in a patient on warfarin should be reversed with four-factor prothrombin complex concentrate for rapid effect. Vitamin K can be given but will take time to have any effect, and fresh frozen plasma (FFP) can be considered if four factor concentrate is not available; however, FFP takes longer to administer due to thawing and rate of administration
- hypertonic saline or mannitol can be used emergently to decrease intracranial pressure
- The head of the bed should be elevated to 30 degrees in patients with intracranial hemorrhage, assuming thoracic and lumbar spine precautions are not in effect hyperventilating to regulate cerebral blood flow is of questionable benefit and should only be performed as a temporizing measure

## SIMULATION ASSESSMENT

*Case 1: Fall on Anticoagulation*

- Learner: _________________________________________

***Assessment Timeline***

This timeline is to help observers assess their learners. It allows observer to make notes on when learners performed various tasks, which can help guide debriefing discussion.

**Critical Actions:**

□ Roll patient and suction to avoid aspiration when vomiting
□ Recognize declining GCS and decide to intubate
□ Obtain CT of head
□ Obtain INR and reverse once given result
□ Give either hypertonic saline or mannitol to reduce intracranial pressure

**Summative and formative comments:**

**Milestones assessment:**

## INSTRUCTOR MATERIALS

**Case 2 Title:** Bicycle Accident with Pelvic Instability

**Case Description & Diagnosis (short synopsis):** This is the case of a road cyclist who crashes while going downhill at moderate speed. The patient presents with large scattered abrasions and thigh pain causing reluctance to move the left leg. As the survey progresses, the patient becomes more tachycardic and hypotensive, and learners should pause to obtain a screening pelvic x-ray which reveals an open book pelvic fracture. The team should apply a pelvic binder (either a sheet with towel clips or a commercially available device). Failure to bind the pelvis will result in continued hemodynamic decompensation. The patient’s blood pressure will stabilize but should remain low, prompting recognition of the need for a massive transfusion protocol and intubation.

### Equipment or Props Needed

Simulated patient

Moulage (gauze wrapped around the head, large road rash on extremities)

C-collar

Backboard

Point-of-care ultrasound machine

Simulated monitor

Portable computer for documenter (or equivalent for the specific institution)

Endotracheal tube with stylet

Laryngoscope

Yankauer^TM^ suction

Rapid infuser if available for RN training

Pelvic binder (either towel with towel clips or commercially available device)

### Confederates needed

One emergency medical services (EMS) technician, although this role can be performed by the instructor if desired.

### Stimulus Inventory

**Background and brief information:** The patient arrives on a backboard and with c-collar by EMS to a Level 1 trauma center with one peripheral IV established (taped to arm).

**Initial presentation:** The patient is alert and oriented, has normal vitals, and is complaining of pain to the left thigh and groin.

**How the scene unfolds:** The patient initially arrives stable. If the survey physician assesses pelvic stability, the patient becomes hypotensive, and they should get a pelvic x-ray to identify the open book injury. If they do not assess pelvic stability and proceed through the secondary survey, the patient will remain hemodynamically stable until rolled for back examination, at which point the patient becomes unstable. Once the pelvic injury is identified and the pelvis is bound, the blood pressure stops dropping but will remain low until blood is transfused. If learners do not call for massive transfusion protocol, the blood pressure will transiently improve with crystalloid or single blood transfusion but then begin to drop again until massive transfusion is ordered. If massive transfusion is not ordered, the patient becomes increasingly altered but should not be intubated until properly resuscitated. The case ends once the team arranges for embolization.

### Critical actions

1. Identify unstable pelvis
2. Apply pelvic binder
3. Recognize the need for massive transfusion protocol
4. Intubate patient for declining mental status
5. Arrange for embolization of unstable pelvic fracture

**Case 2 Title:** Bicycle Accident with Pelvic Instability

**Chief Complaint:** road bike accident

**EMS Report:** EMS report: 35-year-old male/female who was riding a road bike downhill at approximately 25 miles per hour when they lost control and crashed. The patient was helmeted and had a loss of consciousness. He/she arrives complaining of pain in the legs where there is a lot of road rash. There is also blood in the hair. He/she received morphine en route.

(if asked): glucose 96, no known medications or medical problems. No known allergies

**General Appearance:** alert

### Primary Survey

- **Airway:** intact
- **Breathing:** equal breath sounds
- **Circulation:** palpable central and peripheral pulses, no active hemorrhage

### History

- **History of present illness:** none known other than EMS report
- **Past medical history:** none
- **Past surgical history:** none
- **Medications:** none
- **Allergies:** none
- **Social history:** denies drugs or alcohol, married with 2 children
- **Family history:** unknown

### Secondary Survey/Physical Examination

- **General appearance:** laying on backboard with pain to left side
- **HEENT:**
  ○ **Head:** gauze head wrap with abrasions but no identified laceration
  ○ **Eyes:** within normal limits
  ○ **Ears:** within normal limits
  ○ **Nose:** dried blood
  ○ **Throat:** within normal limits
- **Neck:** in c-collar, nontender
- **Heart:** normal rate, no murmurs
- **Lungs:** equal breath sounds
- **Abdominal/GI:** left lower quadrant tenderness, nondistended. (if asked, there is movement of the pelvis with lateral compression)
- **Genitourinary:** no gross blood
- **Rectal:** normal rectal tone (may defer rectal exam)
- **Extremities:** normal strength and sensation, no tenderness to palpation or deformities
- **Back:** no thoracic or lumbar tenderness or step offs
- **Neuro:** within normal limits
- **Skin:** large abrasions to left upper and lower extremities
- **Psych:** within normal limits

*Normal Chest Radiograph (CXR)*

Chest x-ray plain film normal. In: Wikimedia Commons. https://commons.wikimedia.org/wiki/File:Chest_x-ray_plain_film_normal.jpg. Published November 20, 2017. Accessed June 22, 2020. CC-BY-SA-4.0.

*Chest radiograph (CXR) showing endotracheal tube in the appropriate position*

Author’s own image

*Open Book Pelvic Radiograph (CXR)*

Nevit. Diastasis symphysis pubis. In: Wikimedia Commons. https://commons.wikimedia.org/wiki/File:Diastasis_symphysis_pubis_1300500.JPG. Published October 12, 2010. Accessed June 22, 2020.

## OPERATOR MATERIALS

### Diagnosis

open book pelvic fracture

### Disposition

to the catheterization suite for embolization

## DEBRIEFING AND EVALUATION PEARLS

### Case 2: Bicycle Accident with Pelvic Instability

**Team Communication** (May use the nurse documenter feedback form [associated content] to help drive these communication and teamwork debriefing points.):

- Ask the group how they felt regarding the communication during the resuscitation? What techniques were effective, and what were not? Pay particular attention to examples of closed loop communication, how the room was directed to proceed through specific tasks, or if anyone was asked to step back or pause if things were getting too chaotic.
- Did everyone understand what was being done during the case? How could the team leader have ensured that everyone was aware of what steps were being taken? This is most often done by the leader taking a pause and summarizing what has happened and what will happen next, especially before critical steps occur.
- Was it clear who was leading the resuscitation? How were the roles identified? Role identification is best done prior to the patient arriving, with the team leader announcing their name and role aloud and asking everyone in the room to do the same. Another helpful tactic is for the team leader to stand at the foot of the bed and not perform any other task during the resuscitation.
- Were the appropriate tasks performed in a coordinated, logical fashion? Why or why not? This can often be difficult during a trauma survey because certain interventions need to be done prior to completion of the survey. As the primary survey moves along from airway, breathing, circulation, disability, and exposure (A-B-C-D-E), any life-saving intervention should be performed once the need is identified and prior to moving on to the next element of the survey. Definitive management of issues identified in the primary survey should take priority before beginning the secondary survey.

#### Medical Management

- Pelvic stability should be assessed during a trauma survey. If instability is noted, subsequent exams should be deferred.
- Pelvic fractures can lead to significant retroperitoneal bleeding due to a large number of bony fragments and shearing against vasculature in the pelvis. Retroperitoneal bleeding may result in a negative FAST.
- Binding of the pelvis should be performed as soon as an unstable pelvic fracture is suspected to minimize bony fragment movement and decrease the potential space for blood to pool.
- Pain management in the hypotensive trauma patient can be difficult because opiate pain medication usually results in some lowering of blood pressure. At the same time, physicians should not ignore significant pain and suffering in trauma victims. Preference should be given to using short acting agents such as fentanyl at the lowest reasonable dose to control pain. Consideration can also be given to using ketamine at pain controlling doses such as 0.25 mg/kg.
- Patients with unstable traumatic pelvic injuries can easily become hemodynamically unstable. There should be a low threshold for massive transfusion, particularly if multiple units of packed red blood cells are expected to be used.
- Hemodynamic instability and mental status changes in a trauma patient are indications for intubation and stabilization of the airway. Especially in the elderly or those taking heart rate controlling medications such as beta blockers, shock may not always manifest with tachycardia, and consideration of other end organ dysfunction such as altered mental status or evaluation of base deficit should be incorporated into decision-making.
- Definitive management of unstable pelvic fractures should be performed with embolization, usually with interventional radiology. Pre-peritoneal packing with acute care surgery teams is an option; however, this should not be the first step in management because it is not preferred.

## SIMULATION ASSESSMENT

*Case 2: Bicycle Accident with Pelvic Instability*

Learner: _________________________________________

***Assessment Timeline***

This timeline is to help observers assess their learners. It allows observer to make notes on when learners performed various tasks, which can help guide debriefing discussion.

**Critical Actions:**

□ Roll patient and suction to avoid aspiration when vomiting
□ Identify unstable pelvis
□ Apply pelvic binder
□ Recognize need for massive transfusion protocol
□ Intubate patient for declining mental status
□ Arrange for embolization of unstable pelvic fracture

**Summative and formative comments:**

**Milestones assessment:**
